# Individual CLA Isomers, c9t11 and t10c12, Prevent Excess Liver Glycogen Storage and Inhibit Lipogenic Genes Expression Induced by High-Fructose Diet in Rats

**DOI:** 10.1155/2015/535982

**Published:** 2015-05-18

**Authors:** Edyta Maslak, Elzbieta Buczek, Antoni Szumny, Wojciech Szczepnski, Magdalena Franczyk-Zarow, Aneta Kopec, Stefan Chlopicki, Teresa Leszczynska, Renata B. Kostogrys

**Affiliations:** ^1^Department of Human Nutrition, Faculty of Food Technology, Agricultural University of Krakow, Balicka 122, 30-149 Krakow, Poland; ^2^Jagiellonian Centre for Experimental Therapeutics (JCET), Jagiellonian University, Bobrzynskiego 14, 30-348 Krakow, Poland; ^3^Department of Chemistry, Faculty of Food Science, Wroclaw University of Environmental and Life Sciences, C. K. Norwida 25, 50-375 Wroclaw, Poland; ^4^Department of Clinical and Experimental Pathomorphology, Jagiellonian University Medical College, Grzegorzecka 16, 31-531 Krakow, Poland; ^5^Department of Experimental Pharmacology, Jagiellonian University Medical College, Grzegorzecka 16, 31-531 Krakow, Poland

## Abstract

This study assessed the effects of individual conjugated linoleic acid isomers, c9t11-CLA and t10c12-CLA, on nonalcoholic fatty liver disease (NAFLD) and systemic endothelial dysfunction in rats fed for four weeks with control or high-fructose diet. The high-fructose diet hampered body weight gain (without influencing food intake), increased liver weight and glycogen storage in hepatocytes, upregulated expression of fatty acid synthase (FAS) and stearoyl-CoA desaturase-1 (SCD-1), and increased saturated fatty acid (SFA) content in the liver. Both CLA isomers prevented excessive accumulation of glycogen in the liver. Specifically, t10c12-CLA decreased concentration of serum triacylglycerols and LDL + VLDL cholesterol, increased HDL cholesterol, and affected liver lipid content and fatty acid composition by downregulation of liver SCD-1 and FAS expression. In turn, the c9t11-CLA decreased LDL+VLDL cholesterol in the control group and downregulated liver expression of FAS without significant effects on liver weight, lipid content, and fatty acid composition. In summary, feeding rats with a high-fructose diet resulted in increased liver glycogen storage, indicating the induction of gluconeogenesis despite simultaneous upregulation of genes involved in *de novo* lipogenesis. Although both CLA isomers (c9t11 and t10c12) display hepatoprotective activity, the hypolipemic action of the t10c12-CLA isomer proved to be more pronounced than that of c9t11-CLA.

## 1. Introduction

The metabolic syndrome (MS) is a cluster of interrelated risk factors that promote the development of cardiovascular disease (CVD). Recently, MS was also found to be a strong predictor of NAFLD, which is widely accepted to be the hepatic manifestation of MS [1, 2]. To date, increased fat consumption is considered the major pathogenic factor of MS and NAFLD. However, recent data show that elevated consumption of fructose may also contribute to the development of those diseases. Studies in humans and experimental animals demonstrate that increased intake of fructose can result in hypertriglyceridemia, insulin resistance, and liver steatosis [3–5]. Moreover, the existence of cross talk between the liver and vascular wall might specifically account for the strong associations between liver steatosis and accelerated atherosclerosis. It has been shown that severe hepatic steatosis was linked to increased atherogenesis in diabetic patients by increased plasma TG, LDL, and VLDL concentrations and decreased HDL level and insulin resistance [6].

CLA comprises a group of positional and geometric isomers of linoleic acid (C18:2, n-6). In contrast to the chain structure of the linoleic acid, in CLA the double bonds are separated by only one single bond and occur most frequently at carbons 9 and 11 as well as 10 and 12 forming c9t11 and t10c12-CLA isomers [7]. Studies have shown that CLA isomers actively inhibit carcinogenesis [8] and prevent atherosclerosis [9], diabetes [10–12], obesity [13, 14], and osteoporosis [15–17]. Recently, it has been shown that CLA can prevent NAFLD induced by a high-fat diet [18] and influence endothelium-mediated vascular homeostasis, by reducing the release of proinflammatory mediators, such as prostaglandin E_2_ and the vasoconstrictive agent thromboxane A_2_, within vascular endothelial cells [19, 20]. However, the effects of individual CLA isomers, c9t11 and t10c12-CLA, on fructose-induced NAFLD and endothelial dysfunction have not been investigated.

## 2. Materials and Methods

### 2.1. Animals and Experimental Design

Male Wistar rats at an initial body weight of approximately 100 g were divided into 6 experimental groups and fed for 4 weeks with either the control AIN-93G diet (C), a high-fructose diet (F, 63% fructose), or C and F diets supplemented with 1% c9t11 and t10c12-CLA isomers (Larodan Fine Chemicals, Malmo, Sweden). The addition of individual CLA isomers was calculated considering the purity of c9t11 (75%) and t10c12 (90%) and balanced at the expense of quantity consisted of soybean oil (Table 1). The control and experimental diets were prepared freshly according to Reeves et al. [21] and stored in darkness at 4°C to avoid lipid peroxidation.

Rats were housed in collective cages (3 rats per cage) in a room with a 12 h light-dark cycle and were given* ad libitum* access to diet and water. Rats were weighed weekly, and leftover food was measured daily for the calculation of food intake.

All procedures involving animals were conducted according to the Guidelines for Animal Care and Treatment of the European Union and were approved by the Local Animal Ethics Commission.

### 2.2. Blood Biochemistry

Blood samples were collected from the heart and centrifuged (1000 ×g for 10 min.) to obtain serum. Serum samples were analysed using commercially available kits for total cholesterol (CHOL), HDL, and TG (Liquick Cor no. 2-211, 2-217, 2-262, resp., Cormay, Lublin, Poland). LDL + VLDL concentration was calculated as the difference between CHOL and HDL concentrations. Alanine aminotransferase (ALT) and aspartate aminotransferase (AST) were measured by commercially available kits (numbers A6624-050 and A6661-050, Alpha Diagnostics, Warsaw, Poland) according to the manufacturer's instructions. Blood glucose concentrations were measured using an Accu-Chek Active glucose meter (Roche Diagnostics GmbH, Mannheim, Germany). Uric acid (UA) was analysed using commercially available kit (no. K6681-050, Alpha Diagnostics, Warsaw, Poland).

### 2.3. Histological Evaluation

Fragments of livers were fixed in 4% buffered formalin and prepared according to the standard paraffin method. Paraffin-embedded 5 *μ*m sections were stained with hematoxylin and eosin (HE) for general histology and the Periodic Acid-Schiff (PAS) method was used for glycogen visualization. Sections were photographed under the 200x magnification with a Nikon Eclipse E400 light microscope equipped with a Nikon digital sight DS-Fi1 camera and NIS-Elements software (Nikon GmbH, Düsseldorf, Germany).

### 2.4. Liver Fat Content

Liver fragments were lyophilized and ground. The determination of fat content was performed with a Foss Tecator Extraction system Soxtec Avanti 2050 (Tecator Foss, Hillerød, Sweden) by continuous extraction of the fat from the material and then evaporating the solvent and drying and weighing the residue.

### 2.5. Liver Fatty Acid Composition

The liver lipid extraction was obtained according to the previously described method [22]. Briefly, the extracted fat was saponified (10 min. at 75°C) with a 0.5 M solution of KOH/MetOH and subjected to methylation (10 min. at 75°C) in 14% (v/v) BF_3_/MetOH (Sigma-Aldrich, St. Louis, MO, USA). Then, the methyl esters of fatty acids were extracted with hexane (Avantor Performance Materials Poland S.A., Gliwice, Poland) and analysed using a gas chromatograph coupled with a mass spectrometer (Shimadzu GCMS QP 5050, Shimadzu, Kyoto, Japan). Separation was achieved using SP-2560 capillary column with a length of 100 m, inner diameter of 0.25 mm, and film thickness of 0.25 *μ*m (Supelco, St. Louis, MO, USA). Helium was the carrier gas.

Identification of methyl esters of long-chain fatty acids was based on the reference standards (FAME Mixture, Larodan Fine Chemicals, Malmo, Sweden) and a library of mass spectra (NIST 1.7). Percentage of methyl esters of fatty acids was calculated from the analytical signal with the formula (*A*_*i*_/∑_*i*_ *A*) · 100 where *A*_*i*_ is the *i*th signal of the ester and ∑_*i*_ *A* is the sum of all identified analytical signal esters [23].

### 2.6. Real-Time qRT-PCR

Total RNA was isolated from liver samples with a “Total RNA” kit (A&A Biotechnology, Gdynia, Poland) according to the manufacturer's instruction and quantified with a NanoDrop 1000 (NanoDrop Technologies, Wilmington, Delaware, USA). Subsequently, samples were purified with an RNeasyMinElute Cleanup kit (Qiagen, Hilden, Germany) and analysed with a BioAnalyzer (Agilent, Santa Clara, California, USA) to measure final RNA quality and integrity.

Expression of the FAS and SCD-1 genes was checked by qRT-PCR with the following primers: 5′-TCGACCTGCTGACGTCTATG-3′ (forward), 5′-TCTTCCCAGGACAAACCAAC-3′ (reverse) and 5′-TCCTGCTCATGTGCTTCATC-3′ (forward), 5′-GGATGTTCTCCCGAGATTGA-3′ (reverse). FAS and SCD-1 mRNA sample concentrations were analysed using a LightCycler (Roche Diagnostics, Basel, Switzerland) with a SYBR-green fluorochrome (Qiagen, Valencia, CA, USA). Results are presented as the ratio of the expression of each gene to *β*-actin expression (*β*-actin primers: 5′-ACATCCGTAAAGACCTCTATGCCAACA-3′ (forward), 5′-GTGCTAGGAGCCAGGGCAGTAATCT-3′ (reverse)).

### 2.7. Assessment of Endothelial Function

Thoracic aortas were carefully dissected out of the surrounding tissues. The assessment of NO-dependent endothelial function in the isolated rings of rat aortas was determined by dose-dependent relaxation to acetylcholine (Ach, 0.01–10 *µ*M, Sigma-Aldrich, St. Louis, MO, USA) both without and in the presence of the inhibitor of nitric oxide synthase N_*ω*_-Nitro-L-Arginine Methyl Ester (L-NAME, 300 *μ*M, Sigma-Aldrich, St. Louis, MO, USA) as previously described [24]. The endothelium-independent response was tested by a response evoked by sodium nitroprusside (SNP, 0.001–1 *µ*M, Sigma-Aldrich, St. Louis, MO, USA).

### 2.8. Statistical Analysis

Data are expressed as means ± SEM. The Shapiro-Wilk test was applied to test the assumption of normality. Based on those results, either a nonparametric Kruskal-Wallis test or two-way analysis of variance (ANOVA) and Duncan post-hoc test were used to assess the statistical significance at a significance level of *P* ≤ 0.05. The results were analysed using STATISTICA 10.0 software.

## 3. Results

### 3.1. Body Weight and Food Intake

**Figure 1:**
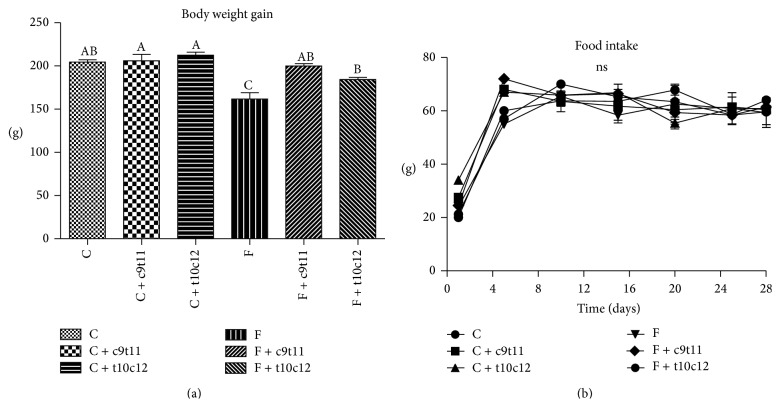
Body weight gain (a) and food intake (b) of rats fed with experimental diets. Values are means ± SEM (*n* = 6). Values with different superscript letters were significantly different (*P* ≤ 0.05).

The final body weight gain of the high-fructose-fed rats (group F) was significantly less than that of rats in group C. The CLA isomers had no effect on body weight in rats fed with diet C, while rats fed with diets F + c9t11 and F + t10c12-CLA gained significantly more weight than rats from group F, reaching weights similar to those of C group (Figure 1(a)). The changes in body weight did not depend on the food intake, which did not differ between experimental groups (Figure 1(b)).

### 3.2. Plasma Biochemistry

No changes in serum lipid profile were observed after four weeks of feeding rats with a high-fructose diet (group F) compared with group C. The addition of the t10c12-CLA isomer decreased LDL + VLDL in C + t10c12 and F + t10c12 groups by 50% and 60%, respectively, and increased HDL concentration by 65% in rats from the F + t10c12 group. In turn, c9t11-CLA decreased LDL + VLDL by 50% only in the group receiving the C + c9t11 diet. No changes were observed in TG concentration between groups C and F; however, TG concentration decreased in the F + t10c12 group compared with the F + c9t11 group and increased in F + c9t11 versus C + c9t11.

Although no differences were observed in serums ALT and AST between groups C and F, addition of c9t11-CLA to a high-fructose diet (F + c9t11 group) significantly decreased ALT concentration in rats compared with rats in the C + c9t11 group. The glucose and UA concentration did not differ between experimental groups (Table 2).

### 3.3. Liver Weight and Fatty Acid Composition

**Figure 2:**
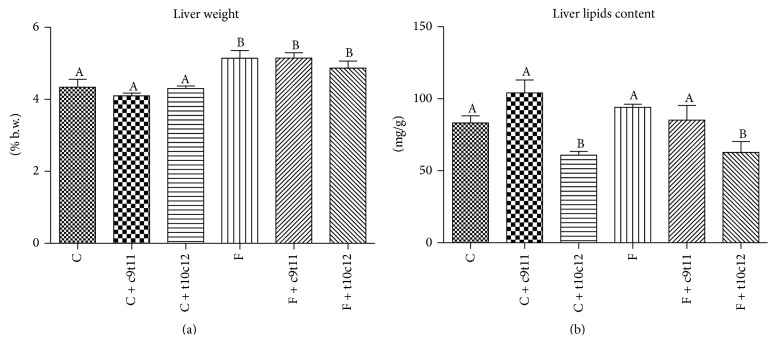
Liver weight (a) and total liver lipids content (b) in rats fed with experimental diets. Values are means ± SEM (*n* = 6). Values with different superscript letters were significantly different (*P* ≤ 0.05).

The high-fructose diet increased liver weight in rats (20% versus control group, *P* < 0.05) in comparison with control group. The CLA isomers (c9t11 and t10c12) had no effect on rats liver weight. Interestingly, the liver lipids content did not differ between groups C and F, while the addition of the t10c12-CLA isomer to diets C and F led to a significant reduction in the liver lipids. The c9t11-CLA isomer had no effects on lipids storage in the rats' livers (Figure 2).

Analysis of liver fatty acid composition revealed that CLA isomers (c9t11 and t10c12) were incorporated into liver lipids, whereas they were not found in the livers of rats fed with control or high-fructose diets (Table 3). Moreover, in C + t10c12 and F + t10c12 groups a small amount of c9t11-CLA isomers was observed.

The high-fructose diet significantly increased total SFA and monounsaturated fatty acids (MUFA) and decreased total polyunsaturated fatty acids (PUFA) in the liver compared with the control group. Among rats fed with a high-fructose diet, addition of the t10c12-CLA isomer increased total SFA and decreased total MUFA (*P* < 0.05), while c9t11-CLA had no effect on SFA, MUFA, nor PUFA level. Moreover, a high-fructose diet significantly increased the 16 : 1 to 16 : 0 ratio in the liver, while addition of t10c12-CLA isomer resulted in a significant decrease in 16 : 1 to 16 : 0 and 18 : 1 to 18 : 0 ratios (Table 3).

### 3.4. Liver Histopathology

**Figure 3:**
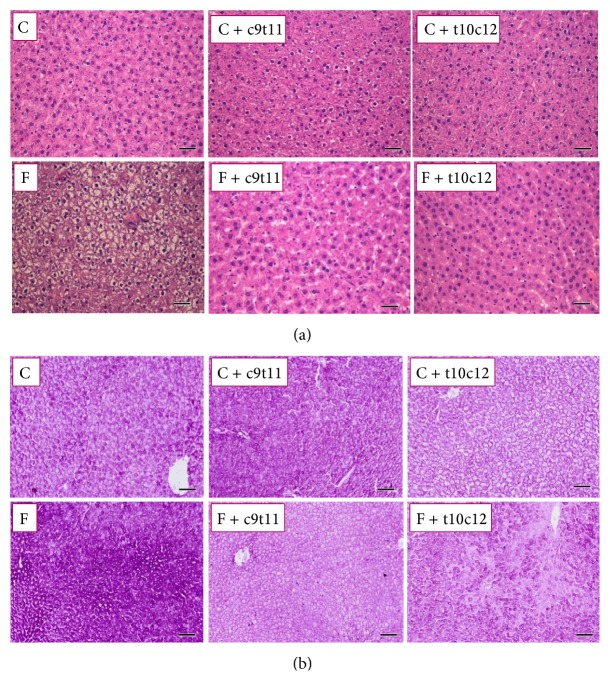
Histopathological images of rat liver samples after four weeks of experimental diets. (a) HE staining. (b) PAS staining. Scale bar = 50 *μ*m.

The high-fructose diet resulted in excessive glycogen storage into hepatocytes without the appearance of steatosis or inflammation, compared with the control group. The CLA isomers, c9t11 and t10c12-CLA, significantly decreased glycogen content in the liver in rats fed with the high-fructose diet (Figure 3).

### 3.5. Gene Expression Analyses (Real-Time PCR)

**Figure 4:**
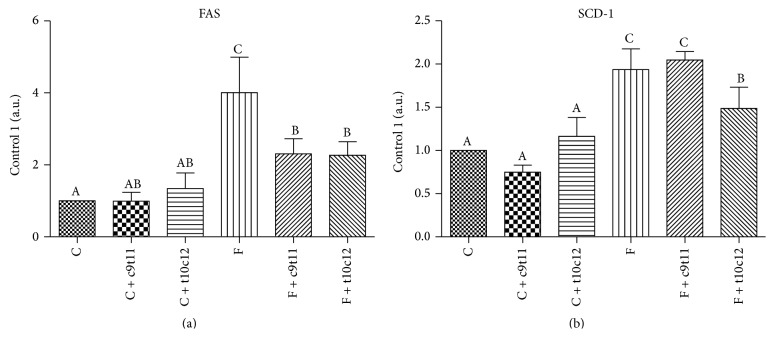
mRNA gene expression of FAS (a) and SCD-1 (b) in rats liver after four weeks of experimental diets. Values are means ± SEM (*n* = 6). Values with different superscript letters were significantly different (*P* ≤ 0.05).

The high-fructose diet increased mRNA expression of FAS and SCD-1 in the liver compared with control animals. The addition of individual isomers to the control diet had no effect on gene expression levels in the liver. Among rats fed with the high-fructose diet, both c9t11 and t10c12-CLA isomers significantly decreased FAS expression, while SCD-1 expression was decreased only in the t10c12-CLA isomer group (Figure 4).

### 3.6. Assessment of Endothelial Function

**Figure 5:**
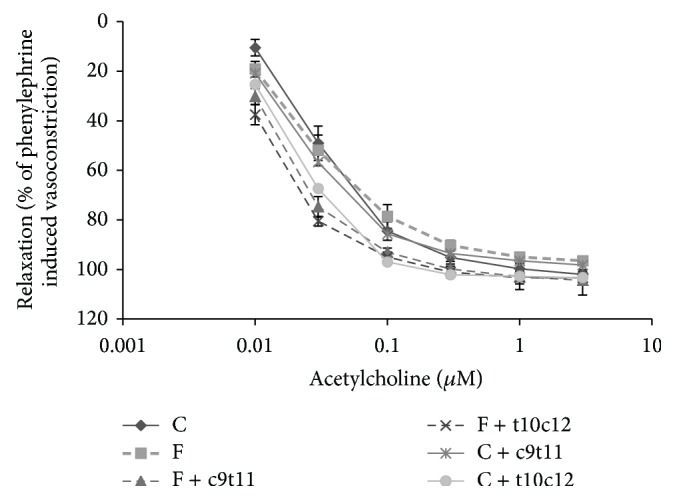
NO-dependent vasodilatation in aortas induced by acetylcholine in rats after four weeks of experimental diets. Values are means (*n* = 6). Values with different superscript letters were significantly different (*P* ≤ 0.05).

The high-fructose diet had no effect on the magnitude of NO-dependent vasodilatation in aorta induced by Ach. The addition of individual CLA isomers (c9t11 and t10c12) to the high-fructose diet resulted in a slightly, but not significantly improved endothelial function observed as an increased NO-dependent vasodilatation at low concentration of Ach (0.03 *μ*M), while no statistically significant difference in Ach-dependent response was at high concentration of Ach (1 *μ*M) (Figure 5). Furthermore, in the presence of L-NAME (300 *μ*M) the Ach-induced vasodilatation in aorta rings was substantially inhibited in all experimental groups. The endothelium-independent response to SNP was not affected by experimental treatment.

## 4. Discussion

In the present study, we found that a high-fructose diet decreased body weight gain without an effect on food intake and induced liver enlargement associated with excessive accumulation of glycogen in hepatocytes. Simultaneously, upregulation of mRNA expression of lipogenic genes, FAS and SCD-1, and changes in liver fatty acids composition were observed. The addition of CLA isomers (c9t11 and t10c12) to the high-fructose diet exhibited hepatoprotective effects manifested as reduced glycogen content in the liver. Moreover, t10c12-CLA, but not c9t11-CLA, decreased the concentration of serum TG (although not significantly) and LDL + VLDL cholesterol and increased HDL level. Additionally, t10c12-CLA reduced liver lipid content and affected fatty acid composition by downregulating liver SCD-1 and FAS expression. Similarly, c9t11-CLA decreased liver mRNA expression of FAS, though it had no significant effect on SCD-1. The slightly improved NO-dependent function in aortas from rats fed with the high-fructose diet and either c9t11 or t10c12 may suggest a vasoprotective effect of CLA isomers, but further studies would be necessary to confirm these findings.

Several studies have shown that high-fructose intake may lead to adverse metabolic alteration, in particular increase in plasma TG, hepatic insulin resistance, and liver steatosis [3, 25]. ALT and AST are commonly used in the assessment of liver function. An increase in ALT activity is often associated with the development of fatty liver, although some authors do not confirm these observations and show that the absence of elevated liver enzymes does not exclude NAFLD diagnosis [26, 27]. In the current work, in which the effect of short-term high-fructose diet was studied, liver enzyme concentrations showed no differences among experimental groups; however, increased liver weight compared with control animals was observed. Some investigators have hypothesized that these effects may be due to increased lipid content in the liver [28–30]. To test this hypothesis, we assessed liver total lipid content and performed histological analysis of the liver samples. The results showed increased glycogen storage in rats fed with the high-fructose diet without a difference in liver fat content between the control and high-fructose groups, indicating the predominance of gluconeogenesis over* de novo* fatty acid synthesis in the early phase response to a high-fructose diet. Interestingly, further analysis of mRNA expression of two lipogenic genes, FAS and SCD-1, exhibited upregulation of both after consuming the high-fructose diet. These observations clearly show that the adverse effect of fructose on glucose metabolism in the liver is closely linked to the alterations in lipid metabolism. Although we did not observe any differences in liver lipid content after the high-fructose diet, we did observe significant alterations in liver fatty acid composition in rats. We found that the high-fructose diet increased liver SFA and MUFA with a simultaneous reduction in PUFA content. Previously, it was shown that the balance between SFA and UFA is a prognostic marker in NAFLD/NASH [31, 32], as well as in cardiovascular and total mortality in humans [33]. As lipids play a crucial role not only in energy storage but also as structural components of cellular membranes and actively participate in cellular signalling, observed in the current study, altered balance between SFA and UFA is of great importance. Furthermore, it has been previously shown that SFA and UFA had distinct impact on cell viability and apoptosis [34]. SFA (palmitic acid (C16:0) and oleic acid (C18:0)) were shown to increase apoptosis of endothelial cells, while UFA (palmitoleic acid (C16:1), oleic acid (C18:1), and linoleic acid (C18:2)) did not promote apoptosis but prevented stearate-induced apoptosis in endothelial cells [34]. Moreover, myotubes' exposure to SFA (C16:0 and C18:0), but not to UFA (C16:1, C18:1, and C18:2), resulted in activation of an inflammatory response due to induction of COX-2 expression and subsequent prostaglandin E_2_ production [35]. In the current study, the fructose-induced alterations in SFA, MUFA, and UFA content were largely due to significant increases in C16:0 and decreases in C18:2 and linolenic acid (C18:3). An increased C16:0 level has been shown to induce accumulation of ceramides leading to a significant decrease in NO generation and insulin resistance [36, 37]. A depletion in NO bioavailability and insulin resistance is associated with liver steatosis [38, 39], suggesting a prominent pathogenetic role of fructose in liver injury. Interestingly, in the current study, the high-fructose diet also increased C16:1 concentration; however, since C16:1 was showed to be, apart from C18:1, the most abundant monounsaturated fatty acid in various kinds of lipids, including phospholipids, triglycerides, cholesterol esters, wax esters, and alkyldiacylglycerols [40], its increased concentration in a state of upregulation of lipogenic genes is not surprising.

In the present work we did not find any effects of high-fructose diet on endothelial function assessed as NO-dependent vasodilation in aortic rings [41]. This is probably due to the relatively short time of high-fructose feeding. Bartuś et al. [42] observed endothelial dysfunction after eight weeks of feeding rats with a high-fructose diet. Quite surprisingly, we found that both individual isomers tend to improve endothelium-dependent responses induced by a low concentration of Ach. As proper endothelial function inhibits leukocyte recruitment, smooth muscle cell proliferation, and development of atherosclerotic plaques [43] and both t10c12 and c9t11-CLA slightly improved endothelial function, these findings may have therapeutic implications. It is notable that improvement was comparable with control and high-fructose groups, as high-fructose diet showed no effect on endothelial function in the current study. Interestingly, the c9t11-CLA isomer reduced ICAM-1 and VCAM-1 expression in endothelium and reduced the adhesion of macrophages to endothelium [44], while the t10c12-CLA isomer activated vascular eNOS in an adiponectin-dependent manner [45]. Obviously, CLA isomers may display vasoprotective activity that warrants further studies.

In the present work, we have shown that both isomers exhibited hepatoprotective properties, although the effects of t10c12-CLA were more pronounced than those of c9t11-CLA. The action of t10c12-CLA, unlike c9t11-CLA, involved improvements not only in liver glycogen storage but also in plasma lipid profile and liver lipid content (some of them in treated rats compared with rats in the high-fructose and control groups). These observations may be partially explained by the increased *β*-oxidation or/and higher hepatic TG secretion induced by t10c12-CLA [46–48]. The involved mechanism may also be linked with inhibition of lipogenesis. In the current study, we showed that t10c12-CLA decreased mRNA expression of liver two lipogenic genes, FAS and SCD-1, in rats. Moreover, as a result, changes in the composition of liver fatty acids were observed. The modulation of liver fatty acid composition by mixture of CLA isomers has been confirmed previously [49]. Intake of t10c12-CLA has also been shown to cause an increase of C18:0 and a commensurate decrease of C18:1 in rat livers [50] and mouse adipocyte cultures [51]. In the current study, we also observed a decrease in 18 : 1 to 18 : 0 and 16 : 1 to 16 : 0 ratios in liver lipids, indicating diminished SCD-1 activity. Thus, downregulation of hepatic FAS and SCD-1 expression may contribute to the mechanism of hepatic lipid content reduction and improvement in lipoprotein profile by the intake of t10c12-CLA. Although the c9t11-CLA isomer induced reduction of FAS expression, its effect was not visible in other measurements, suggesting that its effect on lipid metabolism is modest and probably differs from t10c12-CLA.

Reports on the biological effects of CLA isomers differ among species used in the studies. Results from rats and mice are especially ambiguous. While t10c12-CLA increased TG concentration and induced liver steatosis in mice [52, 53], data from studies on rats, similar to results obtained in the current study, showed protective effects of t10c12-CLA against hyperlipidemia and increased liver weight and lipid content [54]. There are many data in the literature that show important differences in metabolic regulation of lipids among different hamster [55], mice [56], and rat strains [57].

In conclusion, in the current study we showed that a short-term high-fructose diet leads to liver enlargement associated with excessive glycogen storage in hepatocytes. This is accompanied by a simultaneous alteration in liver fatty acid composition and upregulation of genes involved in* de novo* lipogenesis, indicating a predominance of gluconeogenesis over* de novo* fatty acids synthesis in the early phase of liver response to fructose feeding. Although both CLA isomers (c9t11 and t10c12) showed hepatoprotective effects, the hypolipemic action of the t10c12-CLA isomer was more pronounced than that of c9t11-CLA due to its favourable alteration of the plasma lipid profile, decreased liver lipid content, and beneficial changes in liver fatty acid composition. These results are intriguing, and possible effects of CLA on endothelial function are not conclusive and warrant further studies.

## Figures and Tables

**Table 1:** Composition of the experimental diets.

Ingredients^*^ [g/kg]	C	C + c9t11	C + t10c12	F	F + c9t11	F + t10c12
Fructose	—	—	—	**632.486**	**632.486**	**632.486**
Corn starch	532.486	532.486	532.486	—	—	—
Casein	200	200	200	200	200	200
Sucrose	100	100	100	—	—	—
Soybean oil	70	**56.67**	**58.89**	70	**56.67**	**58.89**
Cellulose	50	50	50	50	50	50
Mineral mix	35	35	35	35	35	35
Vitamin mix	10	10	10	10	10	10
Choline bitartrate	2.5	2.5	2.5	2.5	2.5	2.5
t-Butylhydroquinone	0.014	0.014	0.014	0.014	0.014	0.014
**c9t11- CLA**	**—**	**13.33**	—	—	**13.33**	—
**t10c12- CLA**	**—**	—	**11.11**	—	—	**11.11**

^*^The experimental diets ingredients were obtained from Sigma-Aldrich, St. Louis, MO, USA, with the exception of the following: fructose obtained from Biofan, Poland; corn starch obtained from Agrotrade, Poland; and Casein obtained from Kazeina Polska Sp. z o.o., Poland.

**Table 2:** Lipid profile, UA, glucose concentration, and enzyme activities (AST, ALT) in rats fed with experimental diets^*^.

	C	C + c9t11	C + t10c12	F	F + c9t11	F + t10c12
TG [mmol/L]	2.98 ± 0.42^abc^	2.28 ± 0.06^a^	2.08 ± 0.23^a^	3.86 ± 0.43^bc^	4.29 ± 0.64^c^	2.75 ± 0.35^ab^
TCH [mmol/L]	2.09 ± 0.24^ns^	1.78 ± 0.29^ns^	1.98 ± 0.15^ns^	1.84 ± 0.06^ns^	1.85 ± 0.21^ns^	1.98 ± 0.06^ns^
LDL + VLDL [mmol/L]	0.95 ± 0.16^c^	0.49 ± 0.12^ab^	0.49 ± 0.09^ab^	0.86 ± 0.08^bc^	0.64 ± 0.22^abc^	0.36 ± 0.14^a^
HDL [mmol/L]	1.14 ± 0.19^ab^	1.29 ± 0.26^ab^	1.48 ± 0.11^ab^	0.98 ± 0.09^a^	1.21 ± 0.16^abc^	1.62 ± 0.14^b^
UA [mg/dL]	3.41 ± 0.82^ns^	3.56 ± 0.99^ns^	3.48 ± 1.01^ns^	3.33 ± 0.35^ns^	3.46 ± 0.92^ns^	3.57 ± 0.75^ns^
Glucose [mg/dL]	103.00 ± 2.4^ns^	106.25 ± 4.2^ns^	104.33 ± 3.2^ns^	103.00 ± 5.2^ns^	110.25 ± 3.6^ns^	100.80 ± 2.3^ns^
ALT [U/I]	23.22 ± 4.73^ab^	29.40 ± 8.74^b^	24.86 ± 2.28^ab^	13.93 ± 0.75^a^	12.16 ± 3.14^a^	25.51 ± 2.71^ab^
AST [U/I]	49.76 ± 2.62^ab^	44.81 ± 5.26^ab^	61.71 ± 8.95^b^	33.44 ± 4.88^a^	32.45 ± 7.63^a^	44.05 ± 4.45^ab^

^*^Values are means ± SEM (*n* = 6). Values in the same row with different superscript letters were significantly different (*P* ≤ 0.05).

**Table 3:** Liver fatty acid composition of Wistar rats after four weeks of experimental diets.

Fatty acid [%]	C	C + c9t11	C + t10c12	F	F + c9t11	F + t10c12
C14:0	0.68 ± 0.13^a^	1.35 ± 0.26^ab^	1.44 ± 0.23^b^	1.29 ± 0.14^ab^	1.14 ± 0.31^ab^	0.88 ± 0.17^ab^
C16:0	16.85 ± 0.67^a^	24.95 ± 1.14^b^	28.12 ± 2.12^b^	23.16 ± 1.32^b^	25.52 ± 2.92^b^	26.03 ± 0.43^b^
C16:1	2.70 ± 0.54^a^	5.13 ± 0.47^ab^	2.77 ± 0.46^a^	6.61 ± 0.72^b^	5.91 ± 1.72^b^	2.84 ± 0.11^a^
C18:0	7.69 ± 0.65^ab^	6.30 ± 0.81^a^	14.89 ± 1.00^d^	8.79 ± 1.13^ab^	10.41 ± 0.70^bc^	13.34 ± 1.72^cd^
C18:1	21.41 ± 0.53^a^	19.06 ± 1.19^a^	9.15 ± 2.92^b^	25.22 ± 1.82^a^	19.87 ± 3.88^a^	17.37 ± 1.88^a^
C18:2	34.57 ± 2.08^d^	26.90 ± 2.11^c^	25.24 ± 1.19^bc^	18.76 ± 0.30^a^	20.83 ± 1.48^ab^	20.77 ± 1.21^ab^
C18:3	5.72 ± 1.15^a^	2.21 ± 0.29^b^	1.70 ± 0.16^b^	0.99 ± 0.11^b^	1.24 ± 0.10^b^	1.12 ± 0.07^b^
c9t11-CLA	0.00 ± 0.00^a^	2.46 ± 0.44^c^	0.57 ± 0.22^a^	0.00 ± 0.00^a^	1.42 ± 0.36^b^	0.23 ± 0.06^a^
t10c12-CLA	0.00 ± 0.00^a^	0.00 ± 0.00^a^	1.38 ± 0.71^b^	0.00 ± 0.00^a^	0.08 ± 0.03^a^	1.07 ± 0.40^ab^
C20:4	7.22 ± 1.53^a^	9.25 ± 0.80^ab^	11.50 ± 1.10^bc^	9.07 ± 0.86^ab^	10.49 ± 0.82^abc^	13.16 ± 1.48^c^
C22:4	0.99 ± 0.66^a^	0.17 ± 0.03^a^	0.42 ± 0.06^a^	1.13 ± 0.58^a^	0.47 ± 0.11^a^	0.30 ± 0.01^a^
C22:6	2.18 ± 0.43^a^	2.15 ± 0.12^a^	2.82 ± 0.47^a^	4.99 ± 2.80^a^	2.61 ± 0.24^a^	2.89 ± 0.47^a^

16:1/16:0	0.16 ± 0.03^ab^	0.20 ± 0.01^b^	0.10 ± 0.01^a^	0.28 ± 0.02^c^	0.22 ± 0.04^bc^	0.11 ± 0.00^a^
18:1/18:0	2.85 ± 0.26^a^	3.28 ± 0.66^a^	0.60 ± 0.18^b^	3.03 ± 0.46^a^	1.97 ± 0.45^ac^	1.38 ± 0.27^bc^

SFA [%]	25.22 ± 1.19^d^	32.61 ± 0.85^a^	44.46 ± 1.79^c^	33.24 ± 2.17^a^	37.07 ± 3.55^ab^	40.25 ± 2.13^bc^
MUFA [%]	24.11 ± 0.82^a^	24.19 ± 1.58^a^	11.92 ± 2.48^c^	31.82 ± 2.32^b^	25.79 ± 2.28^ab^	20.21 ± 1.84^a^
PUFA [%]	50.68 ± 1.93^c^	43.16 ± 1.89^a^	43.63 ± 0.88^a^	34.94 ± 3.13^b^	37.14 ± 1.34^ab^	39.54 ± 1.87^ab^

^*^Values are means ± SEM (*n* = 6). Values in the same row with different superscript letters were significantly different (*P* ≤ 0.05).
